# Perceptions of Key Informant Health Professionals before implementing tighter glycaemic targets for women with gestational diabetes mellitus in New Zealand

**DOI:** 10.1371/journal.pone.0271699

**Published:** 2022-08-12

**Authors:** Ruth Martis, Julie Brown, Caroline A. Crowther

**Affiliations:** Liggins Institute, The University of Auckland, Auckland, New Zealand; Bradford Institute for Health Research, UNITED KINGDOM

## Abstract

**Background:**

Tighter glycaemic targets may be of benefit for women with GDM and their infants. Barrier and enabler identification prior to implementation of tighter glycaemic targets for women with GDM may support a successful transition.

**Methods:**

A cross-sectional questionnaire survey was conducted among Key Informant Health Professionals in ten hospitals in New Zealand. The survey assessed what was currently working using less tight glycaemic targets; what barriers and enablers were considered likely when introducing tighter glycaemic targets and whether these perceptions differed by health professional groups.

**Results:**

Sixty Key Health Informant Health Professionals completed the survey. When using the lower glycaemic targets, participants considered that women with GDM found the targets easy to use and that collaborative collegial support was effective. No significant barriers were identified. Perceived enablers identified prior to implementation of tighter targets included receiving collegial support (40, 67%), attending education sessions (38, 63%), use of pocket prompt cards (31, 52%), availability of wall charts (25, 42%) and glycaemic target reminder stickers (24, 40%). For health professionals referring into the Diabetes in Pregnancy Service effective communication (50, 83%) was considered important. Perceived barriers were confusion over glycaemic targets use (27 (45%), not being informed of the glycaemic target change (31, 52%), non-involvement with multidisciplinary decisions (29, 48%) and increased difficulty of blood glucose control for women (48, 80%). Overall, barriers and enablers between Health Professional groups did not differ.

**Discussion:**

Key Informant Health Professionals reported effective communication as a key perceived enabler and that woman would find it more difficult to control their blood glucose concentrations. Education sessions, multidisciplinary engagement, wall charts and stickers were considered effective to overcome the perceived barriers. Further research is needed to assess if the barriers perceived were realised and if the perceived enablers supported the implementation of the tighter glycaemic targets effectively.

## Introduction

Gestational diabetes mellitus (GDM) is increasing globally [1]. GDM significantly increases the risk of maternal and infant complications short and long term [2, 3]. Currently in New Zealand 6.2% of pregnant women develop GDM during their pregnancy [4, 5]. Treatments, guided by glycaemic treatment target recommendations, include lifestyle changes and/or medication that aim to reduce or prevent adverse health outcomes associated with GDM [6, 7]. Research has mainly focussed on diagnostic criteria, management of GDM and treatments [8–10]. Recommendations on glycaemic treatment targets vary internationally and are based on consensus due to a lack of high-quality evidence [10–12] with some research recommending tighter glycaemic targets may be of benefit for women with GDM and their infants [13, 14].

There has been a growing interest in the literature of identification of perceived barriers and enablers that are key to guide effective implementation of practice and behaviour change [15, 16]. Translating knowledge into practice needs to be tailored to specific barriers and enablers for clinical practice to change [17, 18]. Only “14% of significant research findings and discoveries ever enter the real-word context” (p. 178) and this is believed to be due to a lack of clear understanding of how to implement the research results without local barrier and enabler identification [19]. The National Institute of Clinical Studies [20] report stated ‘Identifying barriers to evidence uptake’ highlights that there is a lack of information relating to health professionals’ attitudes, beliefs, perceptions and practices. The NICS report recommended that findings from Key Informant Health Professional questionnaire surveys may be one way of informing effective practice change [20]. A Key Informant has been identified as a person who is knowledgeable and experienced about a certain issue or problem, someone who can ‘unlock’ key information [21, 22]. Published studies comparing perceptions of implementation of tighter glycaemic treatment targets for women with GDM between different groups of health care professionals are limited [13, 23].

In 2014 the New Zealand Ministry of Health published clinical practice guideline recommendations for health professionals on the screening, diagnosis and treatment of gestational diabetes in New Zealand [24]. The guideline was developed as there was variation throughout New Zealand in the care provided to women for diagnosis and treatment of GDM. During development of the guideline, diagnosis, glycaemic treatment targets and treatment recommendations were often agreed by the guideline panel by reaching consensus rather than being able to base recommendations on high-quality research evidence. One of the research recommendations made by the guideline panel identified that a further randomised trial was needed to establish optimal glycaemic treatment targets for women diagnosed with GDM [24]. In response to this the TARGET (Optimising Glycaemic Targets for Gestational Diabetes) Trial comparing use of tight to less tight treatment targets was undertaken (Australian New Zealand Trial Registry: ACTRN12615000282583), [25]. This present study, nested within the TARGET Trial, sought the perception of Key Informant Health Professionals at the participating hospitals on barriers and enablers prior to the introduction of tighter glycaemic treatment targets for women with GDM (fasting plasma glucose ≤5.0mmol/L; 1 hour postprandial ≤7.4mmol/L; 2 hours postprandial ≤6.7mmol/L) (Crowther 2018). The less tight glycaemic treatment targets recommended for women with GDM used by most participating hospitals were fasting plasma glucose ≤5.5mmol/L; 1 hour postprandial ≤8.0mmol/L; 2 hours postprandial ≤7.0mmol/L. The researchers wanted to specifically assess the perception of Key Informant Health Professionals who may suggest effective ways to optimise the implementation of the tighter glycaemic treatment targets recommended in the clinical guideline [24] and used in the TARGET trial. This would provide insight into the perceptions of all health disciplines involved who need to support the implementation of tighter glycaemic targets and enable identified barriers and enablers to be addressed before and during the implementation of tighter glycaemic targets. The specific research questions of this study were:

1. What has worked well and not so well using less tight glycaemic treatment targets amongst Key Informant Health Professionals caring for women with GDM?
2. What are Key Informant Health Professionals’ perception for barriers and enablers prior to the implementation of tighter glycaemic targets?
3. Are there differences of enabler and barrier perception prior to implementing tighter glycaemic targets between Key Informant Health Professionals’ groups?

## Materials and methods

### Study design and setting

A cross-sectional questionnaire survey was conducted among Key Informant Health Professionals recruited from the ten hospitals collaborating in the Target Trial from nine DHB’s providing different levels of maternity care and Diabetes in Pregnancy services in New Zealand between February and November 2016 (N = 60). Six DHB’s were geographically spread over the North Island (Northland, Bay of Plenty, Lakes, Hawkes Bay, Taranaki, and Counties Manukau DHB’s) and three DHB’s were in the South Island (Nelson Marlborough, Canterbury, and Southern (two hospitals)). The birth rates for the DHB’s varied with a range from 1386 (Lakes DHB) to 8287 (Counties Manukau DHB) births per annum [26].

The study was approved by the New Zealand Health and Disability Ethics committee (HDEC) Ref. 14/NTA/163, research registration number 1965. Locality agreements were obtained from all DHB’s.

### Study participants

At each of the 10 hospitals one Key Informant from six health professional groups providing care for women with GDM were identified by the Target Trial site investigator. The six health professional groups included: endocrinologist or diabetes physician, obstetrician, clinical nurse specialist: diabetes or diabetes midwife, diabetes dietitian, hospital midwife and community midwife (lead maternity carer (LMC) midwife). The Key Informant Health Professional had to have been associated with the provision of care for women with GDM for at least four months. The identified Key Informants were contacted by the researcher (RM) and informed about the study and their consent sought for participation.

### Study processes

Participants completed a questionnaire survey, designed for this study, before the implementation of tighter glycaemic treatment targets for women with GDM at their hospital. The questionnaire survey comprised 16 questions and included demographic characteristics of the Key Informant Health Professionals, their thoughts relating to what worked well and what was challenging using the less tight glycaemic treatment targets for women with GDM at their hospital. The questionnaire survey asked the participant’s perception of how the implementation to tighter glycaemic treatment targets may work well or be challenging. The tighter glycaemic targets were fasting plasma glucose ≤5.0mmol/L; 1 hour postprandial ≤7.4mmol/L; 2 hours postprandial ≤6.7mmol/L) [25]. After face validity (Health professionals with expert knowledge read through the questions) was applied to the developed survey questionnaire, an assessment followed of the question constructions (are they confusing, double-barrel or leading questions). The questionnaire survey was then piloted with one obstetrician and one diabetes midwife following which a ‘don’t know’ box was added to one question. Questionnaire surveys were conducted face to face. All participants answered all questions.

The administration of the questionnaire survey was linked to the timing of the stepped wedge randomised trial design of the TARGET Trial [25]. Site visits were conducted prior to commencement of the trial when less tight targets were used and prior to changing over to the tighter glycaemic targets.

### Data analysis

The data were entered into an Excel spreadsheet and analysed using Pivot Tables in Microsoft Office Excel 2016. Descriptive statistics and frequencies were used to analyse the Key Informant Health Professionals’ demographics and questionnaire survey responses.

## Results

All 60 Key Health Informant Health Professionals approached gave consent to participate in this questionnaire survey. All participants answered all the questions. Demographic characteristics of the participants showed that the majority were female (83%), the mean time of the Key Informant Health Professionals practising in their profession was 20.4 years (SD 10.2) and the mean years providing care for women with GDM was 13.2 (SD 7.8) (Table 1). While all Key Informant Health Professionals provided care for women with GDM, 46 (76%) indicated that they directly advised or treated women with GDM. Awareness of the less tight glycaemic treatment targets in use was indicated by 54 (90%) Key Informant Health Professionals, with one obstetrician and five community midwives (LMC) indicating no awareness of these (Table 1).

**Table 1:** Demographic characteristics of Key Informant Health Professionals surveyed across 10 hospitals (n = 60)^#^.

Profession	Number of participants	Gender		Years practicing in profession	Years working with women with GDM	Advise or treat women with GDM	Aware of glycaemic treatment targets
		n (%)					
	n (%)	Female	Male	Means ± SD	Means ± SD	Yes n (%)	Yes n (%)
Endocrinologists or diabetes physician	10 (17)	5 (8)	5 (8)	21.3 ± 7.5	16.0 ± 5.6	10 (17)	10 (17)
Obstetrician	10 (17)	5 (8)	5 (8)	20.6 ± 7.03	15.3 ± 7.1	8 (13)	9 (15)
Clinical nurse specialist: diabetes or diabetes midwife	10 (17)	10 (17)	0 (0)	23.4 ± 13.2	10.3 ± 7.3	10 (17)	10 (17)
Diabetes dietitian	10 (17)	10 (17)	0 (0)	19.3 ± 13.5	10.5 ± 7.9	8 (13)	10 (17)
Hospital midwife	10 (17)	10 (17)	0 (0)	22.5 ± 10.0	15.5 ± 10.0	5 (8)	10 (17)
LMC*community midwife	10 (17)	10 (17)	0 (0)	16.3 ± 8.7	12.9 ± 8.2	5 (8)	5 (8)
**Total**	**60 (100)**	**50 (83)**	**10 (17)**	**20.4 ±10.2**	**13.2 ± 7.8**	**46 (76)**	**54 (90)**

^#^All figures are rounded to the nearest whole number/or to the first decimal point.

*LMC (lead maternity carer) in New Zealand provides lead maternity care (is in charge).

This can be either a midwife, an obstetrician, or a general practitioner (GP) https://www.midwife.org.nz/in-new-zealand/contexts-for-practice

### Enablers with use of less tight glycaemic treatment targets

Over half of the Key Informant Health Professionals identified that women with GDM in their care found the lower glycaemic targets easy to adhere to (36, 60%), successfully controlled the woman’s capillary blood glucose (CBG) concentration (36, 60%), and there was collaborative collegial support in using the same glycaemic treatment targets (32, 53%). Study folders and education materials that were available to support and guide the use of the less tight glycaemic treatment targets were considered to be working well by 16 (27%) Key Informant Health Professionals (Table 2).

**Table 2:** Barriers and enablers with use of less tight glycaemic treatment targets amongst Key Informant Health Professionals providing care for women with GDM^#^.

Enablers–What is currently working well?	Number of health professionals N = 60 (%)	Barriers–What is currently challenging?	Number of health professionals N = 60 (%)
Successful control of CBG* concentrations	36 (60)	Poor glucose control	8 (13)
Women found the glycaemic targets easy to adhere to	36 (60)	Women not adhering to the recommended glycaemic targets	13 (22)
Study folder and education materials helpful reminder	16 (27)	Lack of resources	2 (3)
Collaborative collegial support in the use of glycaemic targets	32 (53)	Different treatment thresholds used by different health professionals	7 (12)
No increase in morbidity noted	20 (33)	Confusion over which glycaemic targets should be used since publication of the Ministry of Health guideline	9 (15)
**Others**: (multiple responses possible) Depending on the woman’s honesty x1 Unsure x1 Group sessions x1 Consistent approach among staff x1 Drop-in clinic & small effective team x1 Stickers and wall charts x3 Women attending church-based diet talks x1 Less postprandial hypoglycaemia x1 Less hypoglycaemia events in labour x1	11 (18)	**Others**: (multiple responses possible) Women not willing to engage, not bringing their monitors x2 No family centred approach x1 Unsure x2 Possible slippery slope effect x1 Women vary in their treatment response x1 Health professional not being informed x1 Late antenatal care x1	9 (15)

^#^All figures are rounded to the nearest whole number

*CBG = capillary blood glucose concentration

The questionnaire survey invited participants to add other comments in free text boxes and 11 (18%) opted to do so (Table 2). A range of comments were presented, occurring only one time each in the responses and ranged from groups sessions working well to drop-in clinics, having church-based diet talks and a consistent approach among staff (Table 2). Three (5%) Key Informant Health Professionals commented that glycaemic target reminder stickers and wall charts were helpful reminders for recommending the current less tight glycaemic treatment targets for women with GDM (Table 2).

### Barriers to the use of less tight glycaemic treatment targets

Key Informant Health Professionals identified only minor challenges with the use of the less tight glycaemic treatment targets (Table 2). Poor glucose control was reported by 8 (13%) Key Informant Health Professionals, women not adhering to the recommended glycaemic targets by 13 (22%), different thresholds used by different health professionals by 7 (12%), confusion over which glycaemic targets should be used since the publications of the New Zealand national guideline by 9 (15%) and lack of resources by 2 (3%) (Table 2). Nine (15%) Key Informant Health Professionals opted to add other challenges into the free text boxes. Comments ranged from women not willing to engage, not bringing their glucose monitors (2, 3%), to women varying in their response to the treatment targets (1, 2%), no family care approach (1, 2%), late antenatal care (1, 2%) and health professional not being informed (1, 2%) (Table 2).

### Perceived enablers to implementation of tighter glycaemic treatment targets

The Key Informant Health Professionals reported perceived enablers for staff to implement use of tighter glycaemic treatment targets as attending education sessions for staff (38, 63%), use of small pocket prompt cards (31, 52%), receiving collegial support (40, 67%), regular reminders of the targets in use (26, 43%) and wall posters detailing the targets in use (25, 42%) (Table 3).

**Table 3:** Perceived enables and barriers to implement tighter glycaemic treatment targets among Key Informant Health Professionals providing care for women with GDM^#^.

Perceived Enablers	What may work well implementing tighter targets N = 60 (%)	Perceived Barriers	What may not work well implementing tighter targets N = 60 (%)
**For staff involved using/recommending glycaemic targets**			
Education sessions	38 (63)	Too few staff	16 (27)
Posters	25 (42)	Lack of access to resources to assist change	13 (22)
Regular reminders	26 (43)	Different treatment threshold used by different health professionals	20 (33)
Pocket prompt cards	31 (52)	Confusion over which glycaemic targets to use	27 (45)
Collegial support	40 (67)	Lack of collegial support	12 (20)
PowerPoint presentations	7 (12)		
**Others**: (multiple responses possible) Clinic room wall chart x25 On-line learning modules x1 Stickers x24 Regular up-dates from diabetes team x2 Weekly meetings x1 Diabetes study day/Workshops x2 Newsletter to Community midwives x1 Peer meetings x2 Small multidisciplinary team x3	41 (68)	**Others**: (multiple responses possible) Not being informed of change, lack of communication x5 More clinic appointments needed x1 Huge geographical area x1 Dislike wall charts x1 Using different locums with different ideas x1 Increased workload x1	10 (17)
**For women using the tighter glycaemic targets**			
Easy to accept	10 (17)	More difficult to control CBG*	48 (80)
Believing it is better for the baby	48 (80)	Believing it will harm the baby	6 (10)
Believing it is good for their health	36 (60)	Inability to attend more frequent clinic appointments	17 (28)
Believing they will have a better birth outcome	39 (65)		
**Others**: (multiple responses possible) Information in the woman’s first language x4 Visual information x3 Involve community support x1 Phone reminder for appointments x1 Free pick-up service for clinic appointments x3 Speak hard words x1 Bribes x1 Iwi initiatives x1 Employ Māori diabetes midwives x1	13 (22)	**Others**: (multiple responses possible) Higher insulin usage x4 Increased phone calls needed by diabetic nurse/midwife x3 More frequent clinic appointments x4 Higher induction of labour rate x2 Women not understanding, feeling restricted x4 Extended family has different belief x1 Women not wanting to engage x2 Potential decrease in food intake x3 No free transport provided x1 Parking difficult x1 No visual resources x1 More paid phone interpreters needed x1 Phone interpreters are often men x1 Information only available in English x2 Confusion for women who were on less tight targets with last pregnancy x2	23 (38)
**For wider hospital services**			
Effective communication	41 (68)	In-effective communication	21 (35)
Increase in multidisciplinary engagement	38 (63)	Lack of collegial support	10 (17)
Overall health cost reduction	17 (28)	Overall health cost increases	10 (17)
Increased evidence information dissemination	27 (45)	Lack of resources	20 (33)
**Others**: (multiple responses possible) Will be business as usual x8 Unsure x6 Keep all inpatient services in the loop x2 Inform community pharmacists x1 Multidisciplinary Diabetes study day/workshop for all x2	19 (32)	**Others**: (multiple responses possible) Increase in clinic appointments, more rooms needed x3 More re-admissions and induction of labour, need more beds x3 Effective access to expert advice x1 Visual resources for all x1 Geographical distance, no outreach clinic x1	9 (15)
**LMC**^‡^ **community midwives**			
Effective communication	50 (83)	Ineffective communication	31 (52)
Involvement with multidisciplinary decisions	29 (48)	Non-involvement with multidisciplinary decisions	29 (48)
Effective access to expert advice	18 (30)	Lack of effective access to expert advice	17 (28)
**Others**: (multiple responses possible) Evidenced based information sharing x9 Multi-disciplinary study days x5 Business as usual x6 Financial provision for community midwives to attend specialist’s clinic appointments with the woman x3 Unsure x3 Electronic communication x2	25 (42)	**Others**: (multiple responses possible) LMC community midwife not informed of treatment change x7 Unable to attend specialists’ appointments with the women x4 LMC community midwife not seeing the women because of increased clinic appointments x2	13 (22)

^#^All figures are rounded to the nearest whole number

*CBG: Capillary blood glucose

^‡^LMC (lead maternity carer) in New Zealand provides lead maternity care (is in charge).

This can be either a midwife, an obstetrician, or a GP. https://www.midwife.org.nz/in-new-zealand/contexts-for-practice

For women with GDM who would be using tighter glycaemic targets, Key Informant Health Professionals perceived those women would be more likely to adhere to the tighter treatment glycaemic targets because they would believe that this was better for their baby (48, 80%) and that they would have a better birth outcome (39, 65%) (Table 3). No increase in morbidity was expected by 20 (33%) Key Informant Health Professionals (Table 3).

Hospital services might possibly be impacted by implementing tighter glycaemic treatment targets. Key Informant Health Professionals saw enabling the implementation through effective communication (41, 68%), an increase in multidisciplinary engagement (38, 63%) and through effective information dissemination (27, 45%) (Table 3).

In New Zealand, community midwives, also known as Lead Maternity care (LMC) providers, provide autonomous care for pregnant women in their care, unless a referral or hand-over to specialists’ care is required. Community midwives often continue the care for women with GDM in a shared care arranged with their diabetes and obstetrician colleagues. A change of glycaemic treatment targets may have an impact on community midwives’ provision of care. The two main perceived enablers by Key Informant Health Professionals were effective communication (50, 83%) and involvement with multidisciplinary decisions (29, 48%) (Table 3).

Further enablers were listed by 41 (68%) participants in the free text box who perceived clinic room wall charts (25) and stickers (24) that detailed the treatment targets in use to be the most effective means for successful implementation of tighter glycaemic targets (Table 3) by the staff involved. Stickers would be used for women’s blood glucose recording booklets and for patient notes if documentation was in hard copy form.

For the women, other perceived enablers centred around information to be provided in their own language (4) or visual (3) to enhance the women’s understanding of their glycaemic targets and GDM in general. Having a free pick-up service for clinic appointments was perceived as enabling successful use of tighter glycaemic targets for women with GDM (3).

Most participants perceived no other enablers were needed for the wider hospital services, with ‘business as usual’, as changing to tighter glycaemic treatment targets would not impact on hospital resources (Table 3).

Effective communication, perceived as enablers for community midwives, was further supported with additional comments in the free text box by Key Informant Health Professionals, that included evidenced based information sharing (9), electronic communication (2) and multidisciplinary study days (5) (Table 3). Three participants identified perceived enablers as providing financial support for community midwives to attend specialist’s clinic appointments with the woman (3) (Table 3), to enable effective communication, and everyone being on ‘the same page’.

### Perceived barriers to implementation of tighter glycaemic treatment targets

Participants reported perceived barriers for the implementation of tighter glycaemic targets for staff, women, wider hospital and community midwives (Table 3).

The top two perceived barriers for staff were identified as confusion over which glycaemic targets to use (27, 45%) and different treatment thresholds being used by different health professionals (20, 33%). For the women with GDM, perceived barriers reported were difficulty to control capillary blood glucose (CBG) concentration (48, 80%) and inability to attend more frequent clinic appointments (17, 28%), which would be necessary if the perception of increased difficulty of blood glucose control was realised. Ineffective communication was perceived as a barrier for other hospital services (21, 35%) and community midwives (31, 52%). Lack of resources for the hospital (20, 33%) and non-involvement with multidisciplinary decisions for community midwives (29, 48%) (Table 3) were reported. Conversely these were reflected as perceived enablers by the Key Informant Health Professionals (Table 3).

Other perceived barriers identified was the belief that the information on change to use of tighter glycaemic targets may not be disseminated to all relevant staff (5) (Table 3). For the wider hospital services, the two main perceived barriers were facility provisions, such as a perceived increase in clinic appointments requiring more rooms being available (3) and the perceived increase in re-admissions and induction of labour (3) requiring more beds to be available (Table 3).

The biggest perceived barrier for LMC community midwives centred around not being informed of the treatment target change (7) by the health professionals providing the diabetes care for the pregnant women and the inability to attend specialists’ appointments with the women (4) (Table 3).

### Differences in perceived enablers between Health Professionals prior to implementation of tighter glycaemic targets

Endocrinologists (6, 60%), obstetricians (7 (70%), hospital midwives (7, 70%) and community midwives (10, 100%), most frequently perceived education sessions and collegial support would enable staff to accept, support and implement tighter glycaemic targets (Table 4). While diabetes dietitians and clinical nurse specialist, and diabetes midwives indicated collegial support as well (7, 70%), only 4 (40%) perceived education sessions as enabling effective implementation. There were a range of ‘other’ free text enablers identified (Table 4). All Key Informant Health Professionals, except the community midwives, reported in the free text section, as the most frequent perceived enablers the provision of clinical wall charts and stickers detailing the tighter glycaemic targets (Table 4).

**Table 4:** Perceived enablers prior to implementing tighter glycaemic targets by Key Informant Health Professionals^#^.

Perceived Enablers	Endocrinologist or Diabetes Physician	Obstetrician	Clinical nurse specialist: diabetes or diabetes midwife	Diabetes dietitians	Hospital midwife	LMC^‡^ community midwife
	N = 10 (%)	N = 10 (%)	N = 10 (%)	N = 10 (%)	N = 10 (%)	N = 10 (%)
**For staff involved using glycaemic targets**						
Education session	6 (60)	7 (70)	4 (40)	4 (40)	7 (70)	10 (100)
Posters	7 (70)	5 (50)	3 (30)	4 (40)	1 (10)	5 (50)
Regular reminders	4 (40)	7 (70)	3 (30)	3 (30)	4 (40)	5 (50)
PowerPoint presentations	1 (10)	1 (10)	0 (0)	0 (0)	1 (10)	4 (40)
Pocket prompt cards	6 (60)	3 (30)	4 (40)	1 (10)	2 (20)	4 (40)
Collegial support	9 (90)	5 (50)	7 (70)	7 (70)	6 (60)	6 (60)
**Others** (multiple responses possible)	7 (70)	8 (80)	7 (70)	6 (60)	7 (70)	6 (60)
**Others**: Clinic room wall chart	6 (60)	6 (60)	6 (60)	4 (40)	3 (30)	0 (0)
On-line learning modules	0 (0)	0 (0)	0 (0)	0 (0)	1 (10)	0 (0)
Regular up-dates from diabetes team	0 (0)	0 (0)	0 (0)	0 (0)	1 (10)	1 (10)
Stickers	4 (40)	3 (30)	6 (60)	5 (50)	6 (60)	0 (0)
Weekly meetings	0 (0)	0 (0)	1 (10)	0 (0)	0 (0)	0 (0)
Diabetes study day/Workshops	0 (0)	1 (10)	0 (0)	0 (0)	0 (0)	1 (10)
Newsletter to Community midwives	0 (0)	0 (0)	0 (0)	0 (0)	0 (0)	1 (10)
Peer meetings	0 (0)	1 (10)	0 (0)	0 (0)	0 (0)	1 (10)
Small multidisciplinary team	1 (10)	1 (10)	1 (10)	0 (0)	0 (0)	0 (0)
**For women using the tighter glycaemic targets**						
Easy to accept	1 (10)	3 (30)	2 (20)	1 (10)	2 (20)	1 (10)
Believing it is good for their health	5 (50)	6 (60)	7 (70)	7 (70)	5 (50)	6 (60)
Believing it is better for the baby	10 (100)	7 (70)	8 (80)	8 (80)	8 (80)	7 (70)
Believing they will have a better birth outcome	8 (80)	6 (60)	7 (70)	8 (80)	5 (50)	5 (50)
**Others** (multiple responses possible)	0 (0)	2 (20)	2 (20)	2 (20)	3 (30)	4 (40)
**Others**: Information in the woman’s first language	0 (0)	1 (10)	1 (10)	1 (10)	1 (10)	0 (0)
Visual information	0 (0)	0 (0)	1 (10)	1 (10)	1 (10)	0 (0)
Involve community support	0 (0)	0 (0)	0 (0)	0 (0)	1 (10)	0 (0)
Phone reminder for appointments	0 (0)	0 (0)	0 (0)	0 (0)	0 (0)	1 (10)
Pick up service for clinic appointments	0 (0)	1 (10)	1 (10)	1 (10)	0 (0)	0 (0)
Speak hard words x1	0 (0)	1 (10)	0 (0)	0 (0)	0 (0)	0 (0)
Bribes	0 (0)	0 (0)	0 (0)	0 (0)	0 (0)	1 (10)
Iwi initiatives	0 (0)	0 (0)	0 (0)	0 (0)	0 (0)	1 (10)
Employ Māori diabetes midwives	0 (0)	0 (0)	0 (0)	0 (0)	0 (0)	1 (10)
**For wider hospital service**						
Increased evidence information dissemination	4 (40)	7 (70)	3 (30)	6 (60)	3 (30)	4 (40)
Increase in multidisciplinary engagement	7 (70)	6 (60)	8 (80)	5 (50)	6 (60)	6 (60)
Effective communication	7 (70)	8 (80)	8 (80)	6 (60)	6 (60)	6 (60)
Health cost reduction overall	2 (20)	6 (60)	2 (20)	1 (10)	3 (30)	3 (30)
**Others** (multiple responses possible)	3 (30)	2 (20)	4 (40)	2 (20)	3 (30)	2 (20)
**Others**: Will be business as usual	2 (20)	1 (10)	2 (10)	1 (10)	1 (10)	1 (10)
Unsure	1 (10)	1 (10)	1 (10)	1 (10)	1 (10)	1 (10)
Keep all inpatient services in the loop	1 (10)	0 (0)	1 (10)	0 (0)	0 (0)	0 (0)
Inform community pharmacists	1 (10)	0 (0)	0 (0)	0 (0)	0 (0)	0 (0)
Multidisciplinary Diabetes study day/workshop for all	1 (10)	0 (0)	0 (0)	0 (0)	1 (10)	0 (0)
**For LMC** * **community midwives**						
Effective communication	8 (80)	9 (90)	8 (80)	8 (80)	9 (90)	8 (80)
Involvement with multidisciplinary decisions	4 (40)	6 (60)	8 (80)	4 (40)	3 (30)	4 (40)
Effective access to expert advice	3 (30)	5 (50)	4 (40)	1 (10)	2 (20)	3 (30)
**Others** (multiple responses possible)	4 (40)	5 (50)	3 (30)	5 (50)	3 (30)	5 (50)
**Others**: Evidenced based information sharing	0 (0)	3 (30)	0 (0)	4 (40)	1 (10)	3 (30)
Multi-disciplinary study days	3 (30)	0 (0)	0 (0)	1 (10)	1 (10)	2 (20)
Business as usual	1 (10)	1 (10)	2 (20)	0 (0)	1 (10)	1 (10)
Financial provision for community midwives to attend specialist’s clinic appointments with the woman	0 (0)	2 (20)	0 (0)	0 (0)	0 (0)	2 (20)
Unsure	0 (0)	1 (10)	0 (0)	1 (10)	0 (0)	1 (10)
Electronic communication	0 (0)	0 (0)	1 (10)	1 (10)	0 (0)	0 (0)

^#^All figures are rounded to the nearest whole number

*LMC (lead maternity carer) in New Zealand provides lead maternity care (is in charge).

This can be either a Midwife, an Obstetrician, or a GP. https://www.midwife.org.nz/in-new-zealand/contexts-for-practice

All the endocrinologists (10, 100%) perceived women would respond well to use of tighter glycaemic targets, as women would believe that it will be better for their infants, and similarly perceived by 80% (8) of clinical diabetes nurse specialist or diabetes midwives, hospital midwives and diabetes dietitians (Table 4). Obstetricians and community midwives (both 7, 70%) had a slightly lesser perception of this enabler (Table 4). If women with GDM identify this enabler as well then providing information about the tighter glycaemic targets could be the central message.

Hospital wide perceived enablers were reported as effective communication and an increase in multidisciplinary engagement by all Key Informant Health Professionals. There was a small variation between Key Informant Health Professionals with 80% (8) obstetricians and clinical diabetes nurse specialist, 70% (7) endocrinologists or diabetes physician, 60% (6) diabetes dietitians, hospital midwives and LMC community midwives supporting these enablers. A health cost reduction was only identified as a substantial enabler by the obstetricians (6, 60%) that may affect the wider hospital services (Table 4). There were no other significant perceived enablers noted between Key Informant Health Professionals (Table 4).

Many pregnant women with GDM in New Zealand are cared for by community midwives, who refer the women once diagnosed with GDM to the Diabetes in Pregnancy Service, usually at the nearest tertiary or secondary hospital. Introducing tighter glycaemic targets and any prescribed treatments need to be communicated to the LMC community midwife, who often provides ongoing pregnancy care. In this context it is seems reasonable that effective communication with community midwives was perceived as the highest important enabler among all Key Informant Health Professionals (Table 4). Additionally, obstetricians (6, 60%) and clinical nurse specialists: diabetes or diabetes midwives (8, 80%) perceived that multidisciplinary involvement with decisions were important enablers (Table 4). All identified perceived enablers supported the coming from the ‘same page’ approach.

### Differences in perceived barriers between by Health Professionals prior to implementation of tighter glycaemic targets

There were considerably fewer perceived barriers identified by all Key Informant Health Professionals compared to enablers. The most important perceived barrier identified for staff was the possible confusion over which glycaemic targets to use, by 60% (6) endocrinologists/diabetes physicians and obstetricians, by 50% (5) community midwives, by 40% (4) clinical nurse specialists: diabetes or diabetes midwives and by 30% (3) both by diabetes dietitian and hospital midwives (Table 5). Additionally, diabetes dietitians and community midwives (both 5, 50%) reported different treatment thresholds used by different health professionals was a perceived barrier (Table 5).

**Table 5:** Differences of barrier perception prior to implementing tighter glycaemic targets between Key Informant Health Professionals’ groups^#^.

Perceived Barriers	Endocrinologist or Diabetes Physician	Obstetrician	Clinical nurse specialist: diabetes or diabetes midwife	Diabetes dietitians	Hospital midwife	LMC^‡^ community midwife
	N = 10 (%)	N = 10 (%)	N = 10 (%)	N = 10 (%)	N = 10 (%)	N = 10 (%)
**For staff involved using glycaemic targets**						
Lack of access to resources to assist change	1 (10)	3 (30)	2 (20)	2 (20)	1 (10)	4 (40)
Too few staff	4 (40)	3 (30)	3 (30)	2 (20)	2 (20)	2 (20)
Confusion over which glycaemic targets to use	6 (60)	6 (60)	4 (40)	3 (30)	3 (30)	5 (50)
Different treatment threshold used by different health professionals	2 (20)	4 (40)	1 (10)	5 (50)	3 (30)	5 (50)
Lack of collegial support	1 (10)	3 (30)	1 (10)	0 (0)	3 (30)	4 (40)
**Others** (multiple responses possible)	1 (10)	4 (40)	3 (30)	0 (0)	0 (0)	3 (30)
**Others**: Not being informed of change, lack of communication	0 (0)	2 (20)	0 (0)	0 (0)	0 (0)	3 (30)
More clinic appointments needed	0 (0)	0 (0)	1 (10)	0 (0)	0 (0)	0 (0)
Huge geographical area	0 (0)	0 (0)	1 (10)	0 (0)	0 (0)	0 (0)
Dislike wall charts	0 (0)	1 (10)	0 (0)	0 (0)	0 (0)	0 (0)
Using different locums with different ideas	0 (0)	1 (10)	0 (0)	0 (0)	0 (0)	0 (0)
Increased workload	0 (0)	0 (0)	1 (10)	0 (0)	0 (0)	0 (0)
**For women using the tighter glycaemic targets**						
More difficult to control capillary blood glucose	7 (70)	7 (70)	9 (90)	9 (90)	7 (70)	9 (90)
Believing it will harm the baby	1 (10)	2 (20)	0 (0)	0 (0)	1 (10)	2 (20)
Inability to attend clinic	3 (30)	2 (20)	4 (40)	3 (30)	3 (30)	2 (20)
**Others** (multiple responses possible)	4 (40)	3 (30)	4 (40)	6 (60)	4 (40)	4 (40)
**Others**: Higher insulin usage	4 (40)	0 (0)	0 (0)	0 (0)	0 (0)	0 (0)
Increased phone calls needed by diabetic nurse/midwife	1 (10)	1 (10)	1 (10)	0 (0)	0 (0)	0 (0)
More frequent clinic appointments	2 (20)	0 (0)	1 (10)	1 (10)	0 (0)	0 (0)
Higher induction of labour rate	0 (0)	0 (0)	0 (0)	0 (0)	2 (20)	0 (0)
Women not understanding, feeling restricted	0 (0)	1 (10)	1 (10)	0 (0)	1 (10)	1 (10)
Extended family has different belief	0 (0)	1 (10)	0 (0)	0 (0)	0 (0)	0 (0)
Women not wanting to engage	0 (0)	0 (0)	0 (0)	1 (10)	0 (0)	1 (10)
Potential decrease in food intake	0 (0)	0 (0)	0 (0)	3 (30)	0 (0)	0 (0)
No free transport provided	0 (0)	0 (0)	0 (0)	0 (0)	1 (10)	0 (0)
Parking difficult	0 (0)	0 (0)	1 (10)	0 (0)	0 (0)	0 (0)
No visual resources	0 (0)	0 (0)	0 (0)	1 (10)	0 (0)	0 (0)
More paid phone interpreters needed	1 (10)	0 (0)	0 (0)	0 (0)	0 (0)	0 (0)
Phone interpreters are often men	0 (0)	0 (0)	1 (10)	0 (0)	0 (0)	0 (0)
Information only available in English	0 (0)	0 (0)	0 (0)	0 (0)	0 (0)	2 (20)
Confusion for women who were on less tight targets with last pregnancy	1 (10)	0 (0)	0 (0)	1 (10)	0 (0)	0 (0)
**For wider hospital service**						
Lack of collegial support	1 (10)	2 (20)	3 (30)	0 (0)	2 (20)	2 (20)
In-effective communication	3 (30)	5 (50)	3 (30)	4 (40)	3 (30)	3 (30)
Lack of resources	4 (40)	3 (30)	2 (20)	3 (30)	3 (30)	5 (50)
Overall health costs increase	2 (20)	0 (0)	1 (10)	2 (20)	1 (10)	4 (40)
**Others** (multiple responses possible)	0 (0)	2 (20)	1 (10)	3 (30)	2 (20)	3 (30)
**Others**: Increase in clinic appointments, more rooms needed	0 (0)	0 (0)	1 (10)	2 (20)	0 (0)	0 (0)
More re-admissions and induction of labour, need more beds	0 (0)	2 (20)	0 (0)	0 (0)	0 (0)	1 (10)
Effective access to expert advice	0 (0)	0 (0)	0 (0)	0 (0)	1 (10)	0 (0)
Visual resources for all	0 (0)	0 (0)	0 (0)	1 (10)	0 (0)	0 (0)
Geographical distance, no outreach clinic	0 (0)	1 (10)	0 (0)	0 (0)	0 (0)	0 (0)
For LMC* **community midwives**						
In-effective communication	5 (50)	8 (80)	4 (40)	5 (50)	4 (40)	5 (50)
Non-involvement with multidisciplinary decisions	4 (40)	5 (50)	6 (60)	3 (30)	5 (50)	6 (60)
Lack of effective access to expert advice	2 (20)	3 (30)	2 (20)	3 (30)	4 (40)	3 (30)
**Others** (multiple responses possible)	2 (20)	1 (10)	1 (10)	4 (40)	3 (30)	5 (50)
**Others**: LMC Community midwife not informed of treatment change	1 (10)	0 (0)	1 (10)	2 (20)	1 (10)	5 (50)
Unable to attend specialists’ appointments with the women	1 (10)	1 (10)	0 (0)	1 (10)	1 (10)	0 (0)
LMC community midwife not seeing the women because of increased clinic appointments	0 (0)	0 (0)	0 (0)	1 (10)	1 (10)	0 (0)

For women with GDM, the major perceived barrier by the Key Informant Health Professional was the potential increase in difficulty of controlling their blood glucose concentrations. Ninety percent (9) of the clinical nurse specialists: diabetes or diabetes midwives, the diabetes dietitians and community midwives reported this perceived barrier and 70% (7) each of the endocrinologists/diabetes physicians, obstetricians and hospital midwives Key Informant Health Professionals (Table 5). Only the endocrinologists/diabetes physicians (4, 40%) reported higher insulin usage under ‘others’ as a perceived barrier (Table 5). Endocrinologists/diabetes physicians are usually the initiator of insulin treatment when indicated for women with GDM in New Zealand. Further prescriptions are then usually followed up with the clinical nurse specialists: diabetes or the diabetes midwives, who did not report this as a perceived barrier.

Great variations with low percentage results were identified between Key Informant Health Professionals reporting perceived barriers for the wider hospital services (Table 5). Results above 50% (5) include endocrinologists or diabetes physicians who reported perceived barriers for in-effective communication across hospital services and community midwives (5, 50%) perceiving lack of resources (Table 5).

In-effective communication was reported as a perceived barrier not only for hospital services but for community midwives (Table 5). Over half of the obstetricians (8, 80%), endocrinologists or diabetes physicians (5, 50%), diabetes dietitian (5, 50%) and community midwives (5, 50%) reported this barrier perception, closely followed by 40% (4) each of the clinical nurse specialists: diabetes or diabetes midwives and the hospital midwives (Table 5). All Key Informant Health Professionals groups identified non-involvement with multidisciplinary decisions as a perceived barrier to varying degrees. Sixty percent (6) of clinical nurse specialists: diabetes or diabetes midwives and of community midwives and 50% (5) of obstetricians and hospital midwives perceived this as a potential barrier (Table 5). Community midwives were the only Key Informant Health Professionals who reported significantly on other perceived barriers with 50% (5) identifying not being informed of treatment change (Table 5).

## Discussion

This study was seeking to answer three research questions by identifying current enablers and barriers and the perception to implementing tighter glycaemic targets for women with GDM from Key Informant Health Professionals involved in care for women diagnosed with GDM. The results aimed to inform the implementation of tighter glycaemic treatment targets prior to the commencement of the TARGET Trial [25].

1. **What has worked well and not so well using less glycaemic treatment targets amongst Key Informant Health Professionals caring for women with GDM?**
  When using the lower glycaemic treatment targets participants considered that women with GDM found the targets easy to use and that collaborative collegial support was effective. No significant barriers were identified. In the free text option, stickers and wallcharts were identified as working well. These enablers were identified in multiple areas in the survey and would benefit consideration to use when implementing tighter glycaemic treatment targets for women with GDM.
2. **What are Key Informant Health Professionals’ perception for barriers and enablers prior to the implementation of tighter glycaemic targets?**
  The second research question was analysed in subsections for easier perceived enabler and barrier identification from different GDM care providers and services.
  - **For staff involved in using/recommending glycaemic targets**
    Similar to the findings on the use of less tight glycaemic targets, participants identified collegial support as the main perceived enabler, closely followed by education sessions and pocket prompt card for health professionals involved with the care of women with GDM. Additionally, over half of the participants who opted to answer in the free text option section commented that clinic wall charts and stickers detailing the tighter glycaemic targets would be seen as enabling. Nearly half of the participants identified confusion over which glycaemic targets to use as a perceived barrier, with an additional third of participants perceiving that different treatment thresholds would be used by different health professionals. The identified perceived enablers would potentially address these barriers and can be used to guide the implementation of tighter glycaemic treatment targets for women with GDM.
  - **For women using the tighter glycaemic targets**
    While the less tight glycaemic targets were identified as working well, it was perceived by most participants that tighter glycaemic targets would be more difficult to achieve for women with GDM. Despite this concern, the majority of Key Informant Health Professionals perceived that the tighter glycaemic targets would be acceptable to women with GDM, as women would believe these targets would be better for the baby and their own health, as well as women would believe that they would have better birth outcomes. These findings indicate that women with GDM benefit from receiving clear information, but a study to explore women’s views on enablers and barriers to tighter glycaemic targets is warranted.
  - **For wider hospital services**
    Over two thirds of Key Informant Health Professionals perceived effective communication and increased multidisciplinary engagement would enable the implementation of tighter glycaemic successfully. There was a small perceived concern about needing more clinic rooms if women required more antenatal appointments and more hospital beds for admissions as there may be a higher induction of labour with tighter glycaemic target recommendations. Although ‘business as usual’ was perceived by nearly half of the participants who opted to enter further perceived enablers in the other free text box, a third of participants perceived an overall health cost reduction, whilst a tenth of participants were concerned about a perceived overall health cost increase. Cost-benefit analysis and resource demand when changing to tighter glycaemic treatment targets may be an area to consider when planning to address potential enablers and barriers.
  - **For LMC**, **community midwives**
    Effective communication was the most significant perceived enabler for LMC community midwives, followed by involvement with multidisciplinary decisions. This was conversely reflected in the perceived barrier identification by participants for tighter glycaemic targets implementation identified as in-effective communication and non-involvement with multidisciplinary decisions. Communication and multidisciplinary engagement have been shown to be effective tools for behaviour change and implementation of clinical recommendations and skills [27].
3. **Are there differences of enabler and barrier perception prior to implementing tighter glycaemic targets between Key Informant Health Professionals’ groups?**
  The third research question attempted to elicit if the health professional groups providing care and services for women with GDM identify different significant enablers and barriers as these results may be useful for targeting different professional groups with different implementation strategies for a change over to tighter glycaemic targets for women with GDM.
  - **For staff involved using glycaemic targets**
    Different Key Informant Health Professionals groups identified different perceived enablers for staff involved with glycaemic treatment targets. Education sessions were perceived as important enablers to implement tighter glycaemic targets for women with GDM by all community midwives, over half of the hospital midwives, obstetricians and endocrinologists or diabetes physicians. For clinical nurse specialists, diabetes or diabetes midwives and hospital dieticians this was not a significant enabler. Collegial support was identified by all Key Informant Health Professionals as an enabler for the implementation of tighter glycaemic targets, with the highest indicator from diabetes dieticians and clinical nurse specialists: diabetes or diabetes midwives and endocrinologists or diabetic physicians. What collegial support means for each of the health professional groups was not clear and warrants further research.
    Regular reminders and pocket prompt cards were identified by the majority of the obstetricians and community midwives and by over half of the endocrinologists or diabetic physicians. Except for the community midwives, all other Key Informant Health professionals, commented additionally in the free text section for perceived enablers the provision of clinical wall charts and stickers listing the tighter glycaemic targets.
    PowerPoint presentations were not seen as enabling by any of the Key Informant Health Professionals. While the barrier identification did not identify any significant areas, half of the endocrinologists, obstetricians and community midwives anticipated confusion amongst health professionals over which glycaemic targets to use. These results indicate the importance of supporting different health professional groups with different resources to enable them to implement the tighter glycaemic treatment targets for women with GDM.
  - **For women using the tighter glycaemic targets**
    The majority of Key Informant Health Professionals perceived that woman diagnosed with GDM would not find it easy to accept the tighter glycaemic targets, as it would be more difficult to control capillary blood glucose concentrations. This did not differ significantly across all Key Informant Health Professionals groups. It was perceived that women would manage this challenge, as once women with GDM were provided with in depth information to understand the reason for tighter glycaemic targets, they would believe it was good for their health, better for their infant and that they would have better birth outcomes. While these beliefs were enablers identified highly across all Key Informant Health Professionals, all endocrinologists or diabetes physicians had this perception. Nearly half of the clinical nurse specialist, diabetes or diabetes midwives perceived that tighter glycaemic target would require more frequent clinic visits and perceived this as a barrier, as women with GDM may not be able to attend increased clinic appointments. This was of low concern for all other Key Informant Health Professional groups. Health literacy and access to health services was always a concern and is an area that would need careful attention when implementing tighter glycaemic targets.
  - **For wider hospital service**
    Over half of the obstetricians and diabetes dieticians perceived that implementing tighter glycaemic targets would enable an increase of evidence information dissemination for the wider hospital service. All Key Informant Health Professionals perceived an increase in multidisciplinary engagement across the hospital and significantly identified effective communication as an enabler and ineffective communication by half of the obstetricians. Lack of resources for the wider hospital was perceived by over half of the LMC community midwives but was not defined what this may be for the wider hospital services.
  - For LMC* community midwives
    Effective communication for LMC community midwives were identified as significant perceived enablers by all Key Informant Health Professionals. Half of the obstetricians perceived that LMC community midwives need effective access to expert advice and involvement with multidisciplinary decisions. Clinical nurse specialist, diabetes or diabetes midwife perceived significantly that LMC community midwives need involvement with multidisciplinary decisions. These results corresponded with the perceived barriers response from all Key Informant Health Professionals of ineffective communication and non-involvement with multi-disciplinary decisions. Half of the LMC community midwives opted to comment in the free text identifying a perceived barrier of community midwives not being informed of treatment change. These results highlight the importance of clear communication and multidisciplinary involvement especially for LMC community midwives who usually continue to be involved with antenatal care.

The survey questionnaire results have contributed to the understanding of barriers and enablers perception by Key Informant Health Professionals providing care for women diagnosed with GDM. The overarching perceived barriers and enablers by participating Key Informant Health Professionals identified the importance of regular and clear communication and multidisciplinary health professional involvement when implementing change to use of tighter glycaemic targets and the perception that women diagnosed with GDM may find it more difficult to control their blood glucose concentrations.

There is currently a lack of international studies and in New Zealand addressing enabler and barrier identification from a Health professional perspective for implementing any glycaemic treatment targets for women with GDM. Previous studies within the diabetes field from Australia and the USA, not specifically in women with GDM, have reported sub-optimal communication and lack of professional relationships between health professionals as barriers to effective implementation when change is required [28, 29]. A qualitative study with 18 health care professionals in the Netherlands, who were providing diabetic care, not for women with GDM, explored perception of barriers and enablers for diabetes care [30, 31]. Health professionals perceived multidisciplinary collaboration was effective with clear communication. A survey conducted with 10 World Diabetes Foundations who supported GDM projects in low and middle-income countries (LMIC) identified one of the major barriers for effective GDM services as lack of clear communication about standard protocols in particular about referrals and follow-up care [16]. Identifying potential enablers and addressing potential barriers prior to implementing health care changes is supported in the literature as an effective way to achieve enduring change [17, 20, 32].

Key Informant Health Professionals in this study reported simple, inexpensive enablers that may support the perceived communication concerns and the desired multidisciplinary approach. Education sessions, multidisciplinary engagement, wall charts and glycaemic target reminder stickers were perceived to be effective to overcome the perceived barriers. Further investigation is needed after the implementation of the tighter glycaemic targets to assess if the perceived enablers supported the implementation of tighter glycaemic targets effectively.

No perceived enablers were identified by Key Informant Health Professionals of the identified barrier perception that women diagnosed with GDM will find it more difficult to control their blood glucose concentrations. As this was reported by the majority of participants the question arises if this message is conveyed to women diagnosed with GDM at their first clinic appointment. This could have a potential negative impact on women’s attitude towards understanding and accepting their glycaemic targets. Clinician’s attitude, beliefs and knowledge about diabetes can influence diabetes management and patient’s perception [29]. A recent small mixed method study with women diagnosed with GDM and health professionals providing care for women with GDM in Singapore highlighted the lack of effective communication and access to understandable information or reminders for women with GDM [33]. That study recommended to explore if a GDM-focused smartphone app integrating reminders of glycaemic targets, test results, in depth information, support for dietary and physical exercise interventions are a feasible and acceptable intervention to overcome the identified barriers for women with GDM and health professionals [33]. This may provide consistent communication and information for women with GDM, and health professionals involved in providing care.

The strength of the current study is the survey of Key Informant Health Professionals across six professional groups involved in gestational diabetes care at 10 hospitals across New Zealand with a 100% response rate. Given the lack of research in this area in New Zealand and internationally, this study is exploratory and encourages further follow-up research. Often studies single out one health care provider group. Women diagnosed with GDM have care provided by several health professionals groups. Therefore, it is important to identify collectively and individually from involved health professional groups what barriers or enablers are perceived before implementing tighter glycaemic targets. This can inform effective strategies to enable the implementation of change collectively or with a health professional specific approach. This will enhance effective care for women with GDM.

A limitation of the current study is the small sample size, therefore the study findings cannot be generalised and applied to all New Zealand Diabetes in Pregnancy Services and their involved health professionals. Accessing all health professionals providing care for women with GDM at all New Zealand hospitals was too difficult, hence access to Key Informants was considered the best option at the ten hospitals who consented to participate in the Target Trial. It is recognised that women who have been diagnosed with GDM need to be included into research about enablers and barriers to implementing tighter glycaemic targets. As part of the TARGET study this has been conducted and is published elsewhere [34, 35].

## Conclusions

Key Informant Health Professionals reported effective communication and multidisciplinary health professional involvement as key perceived enablers. A perceived barrier for women with GDM was identified as women would find it more difficult to control their capillary blood glucose concentrations, but that women will believe it is better for their health and the health of their infant. Education sessions for staff and women, multidisciplinary engagement, wall charts and stickers were perceived to be effective to support the identified enablers and to overcome the perceived barriers. This study identifies that an effective strategy for implementing tighter glycaemic treatment targets for women with GDM needs to be carefully planned. Further research is needed after the implementation of the tighter glycaemic treatment targets for women diagnosed with GDM to assess if the barrier perceptions were realised and if the perceived enablers supported the implementation effectively.

## Supporting information

S1 File The survey template is available with this publication. (PDF)

## Abbreviations

CBG: Capillary Blood Glucose
DHB: District Health Board
GDM: Gestational Diabetes Mellitus
LMC: Lead Maternity Carer
