# Supplementary material for: Perceptions of Key Informant Health Professionals before implementing tighter glycaemic targets for women with gestational diabetes mellitus in New Zealand
Source: PLoS One. 2022 Aug 12;17(8):e0271699. doi: 10.1371/journal.pone.0271699 (PMC9374239; doi:10.1371/journal.pone.0271699)
Supplement: S1 File — (PDF) [file pone.0271699.s001.pdf]

Optimal glycaemic targets for gestational diabetes

TARGET

Key Informant Heath Professionals Survey

Glycaemic Targets for women with GDM - Stage 1

Today's date: ☐☐ / ☐☐ / ☐☐
DD MM YY

For office use only

Study ID: ☐

Study Site: ☐

Thank you for participating with this survey.

This survey is designed to gain an understanding of your thoughts about how the less tight glycaemic targets for women with GDM as part of the TARGET trial have been working. What went well? What were the challenges? Additionally, we would like to find out what you think may be barriers and enablers for implementing the tighter glycaemic treatment targets in the next step of the trial? If you agree, we would like to interview you after the completion of the survey for about 10 minutes to find out how some of the barriers you have identified could be overcome. Thank you.

A. Demographics

1.0 What is your profession?

☐

2.0 How many years have you worked in your profession?

☐☐

2.1 How many years have you been working with women diagnosed with GDM?

☐☐

3.0 Gender: male ◯ female ◯

4.0 What age group do you belong to?

- ◯ 25-34 years
- ◯ 35-44 years
- ◯ 45-54 years
- ◯ 55-64 years
- ◯ 65-74 years

B. Less Tight Glycaemic Treatment Targets

5.0 Do you advise women with GDM on glycaemic treatment targets?

Yes ◯ No ◯

6.0 Are you aware that your hospital is currently using less tight glycaemic targets?

(fasting plasma glucose <5.5mmol/L; 1 hour postprandial <8.0mmol/L; 2 hour postprandial <7.0mmol/L)

Yes ◯ No ◯

7.0 Do you think the less tight glycaemic treatment targets have been used well?

Yes ◯ No ◯ Unsure ◯

7.1 Of all women with GDM at your diabetic service, what percentage do you think have been ADVISED to use the tight glycaemic treatment targets?

◯ 10-25% ◯ 26-49% ◯ 50 – 69% ◯ 70-85% ◯ 85-95% ◯ 96-100% ◯ don't know

8.0 What has worked well when using the less tight glycaemic treatment targets? (tick all that apply)

◯ successful control of capillary blood glucose concentrations

◯ women found the glycaemic targets easy to adhere to

◯ study folder and education materials helpful reminder

◯ collaborative collegial support in the use of the glycaemic targets

◯ no increase in morbidity noted

◯ others - Please specify:

8.1 What has not worked well when using the less tight glycaemic treatment targets? (tick all that apply)

◯ poor glucose control

◯ women not complying with the glycaemic targets

◯ different treatment thresholds used by different health professionals

◯ lack of resources

◯ confusion over which glycaemic targets should be used since the trial started

◯ others - Please specify:

C. Tighter Glycaemic Treatment Targets

Your hospital may be randomised to change over to the recommended tighter glycaemic treatment targets:

fasting plasma glucose ≤5.0mmol/L; 1 hour postprandial ≤7.4mmol/L; 2 hour postprandial ≤6.7mmol/L

We are interested in your views on the implementation of the tighter glycaemic treatment targets and what you think may WORK WELL for:

9.0 Staff involved using glycaemic treatment targets? (tick all that apply)

- ◯ education sessions
- ◯ posters
- ◯ regular reminders
- ◯ PowerPoint presentations
- ◯ pocket prompt cards
- ◯ collegial support
- ◯ others - Please specify:

10.0 Women using the tighter glycaemic treatment? (tick all that apply)

- ◯ easy to accept
- ◯ believing it is good for their health
- ◯ believing it is better for the baby
- ◯ believing they will have a better birth outcome
- ◯ others - Please specify:

11.0 For the wider hospital services e.g. obstetric and dietetic service, pharmacy, laboratory or clinic appointments? (tick all that apply)

- ◯ increased evidence information dissemination
- ◯ increase in multidisciplinary engagement
- ◯ effective communication
- ◯ overall health costs reduction
- ◯ others - Please specify:

12.0 LMC community midwives? (tick all that apply)

- ◯ effective communication
- ◯ involvement with multidisciplinary decisions
- ◯ effective access to expert advice
- ◯ others - Please specify:

We are interested in your views on the implementation of the tighter glycaemic treatment targets and what you think may NOT WORK so well for:

13.0 Staff involved using tighter glycaemic treatment targets? (tick all that apply)

- ◯ lack of access to resources to assist the change
- ◯ too few staff
- ◯ confusion over which glycaemic targets to use
- ◯ different treatment threshold used by different health professionals
- ◯ lack of collegial support
- ◯ others - Please specify:

14.0 Women using the tighter glycaemic treatment targets? (tick all that apply)

- ◯ more difficult to control capillary blood glucose concentrations
- ◯ believing it will harm the baby
- ◯ inability to attend clinic appointments
- ◯ others - Please specify:

15.0 The wider hospital service e.g. obstetric and dietetic service, pharmacy, laboratory or clinic appointments? (tick all that apply)

- ◯ lack of collegial support
- ◯ in-effective communication
- ◯ lack of resources
- ◯ overall health costs increase
- ◯ others - Please specify:

16.0 Lead Maternity Carer (LMC) community midwives? (tick all that apply)

- ◯ in-effective communication
- ◯ non-involvement with multidisciplinary decisions
- ◯ lack of effective access to expert advice
- ◯ others - Please specify:

Thank you for completing the survey.
